# Multivariate and Online Prediction of Closing Price Using Kernel Adaptive Filtering

**DOI:** 10.1155/2021/6400045

**Published:** 2021-12-17

**Authors:** Shambhavi Mishra, Tanveer Ahmed, Vipul Mishra, Manjit Kaur, Thomas Martinetz, Amit Kumar Jain, Hammam Alshazly

**Affiliations:** ^1^School of Engineering and Applied Sciences, Bennett University, Greater Noida 201310, India; ^2^School of Electrical Engineering and Computer Science, Gwangju Institute of Science and Technology, Gwangju 61005, Republic of Korea; ^3^Institute for Neuro- and Bioinformatics, University of Lübeck, Lübeck 23562, Germany; ^4^Institute for Manufacturing, Cambridge University, Cambridge, UK; ^5^Faculty of Computers and Information, South Valley University, Qena 83523, Egypt

## Abstract

This paper proposes a multivariate and online prediction of stock prices via the paradigm of kernel adaptive filtering (KAF). The prediction of stock prices in traditional classification and regression problems needs independent and batch-oriented nature of training. In this article, we challenge this existing notion of the literature and propose an online kernel adaptive filtering-based approach to predict stock prices. We experiment with ten different KAF algorithms to analyze stocks' performance and show the efficacy of the work presented here. In addition to this, and in contrast to the current literature, we look at granular level data. The experiments are performed with quotes gathered at the window of one minute, five minutes, ten minutes, fifteen minutes, twenty minutes, thirty minutes, one hour, and one day. These time windows represent some of the common windows frequently used by traders. The proposed framework is tested on 50 different stocks making up the Indian stock index: Nifty-50. The experimental results show that online learning and KAF is not only a good option, but practically speaking, they can be deployed in high-frequency trading as well.

## 1. Introduction

Prediction has applications in a multitude of areas such as economics [1], business planning and production [2], and weather forecasting [3]. However, accurately predicting the value of a variable is one of the very basic and nontrivial problems of the literature. In this article, we focus our attention on financial time-series prediction and its application to stock price forecasting. Stock market is often considered as a chaotic [4], complex [5], volatile [6], and a dynamic mixture of forces driving the movement of a stock. Undoubtedly, its prediction is one of the significant challenges of the literature [7]. Moreover, the Efficient Market Hypothesis [8] states that stock prices reflect all current information, and any new information leads to unpredictability in stock prices. Naturally, significant work has been done in this area. Nevertheless, research clearly specifies that prediction of stocks, especially the nonlinear and nonstationary financial time-series forecasting, is still challenging [9]. In this regard, several models have been developed; for instance, studies focused on volatility [6, 10], option pricing [11], classification of stock movements [12], predicting prices [13], and so on. In addition to this, studies have used a plethora of techniques, for example, support vector machine (SVM) [14], neural network (NN) [15], and genetic algorithm [16]. Nevertheless, a true solution is yet to be found. Moreover, during our literature survey, we found that the paradigm of KAF is not thoroughly investigated. Although, there are a few papers on the topic, e.g., [17, 18], a comprehensive investigation conducted at a large scale eludes the literature. The existing literature focuses on the multiple-kernel learning method and solves different issues such as kernel size and step size. We follow the same line of thought and take the existing methods [17, 19, 20] as the foundation of the proposed work to propose a KAF-based approach for close-price prediction.

We pointed in the previous paragraph that work has ignored KAF as an effective tool for financial time series forecasting. In this context, working with KAF has several advantages. First, it is one most favoured tools of the literature to predict a time series [21, 22]. The techniques in KAF have achieved tremendous accuracy in terms of predictive capability. Second, the convergence speed of KAF-based algorithms is excellent. In other words, they achieve convergence in fewer iterations. Third, they have universal function approximation properties [23]. This has the mathematical property desired for predicting a financial time series. Owing to these reasons, we focus on predicting the financial time series via the paradigm of KAF. Despite these advantages, one of the issues with existing work is Batch Learning. We would argue that Batch Learning is an ineffective tool in financial time-series forecasting. The rationale here is backed by the fact that the data of a financial time series is nonstationary. Therefore, relying on models trained in an offline manner and expecting them to perform well in real market scenarios is a rather strong assumption. To fix this, online learning is proving to be a highly efficient approach [24–27]. In this method, the basis is selected during sample-by-sample training. Moreover, changing circumstances are quickly incorporated, and the algorithm changes its weight vector to make accurate predictions. Hence, we complement the idea of using KAF with online learning to predict a financial time series.

In light of the challenges and the potential solution specified in this section, we propose the paradigm of online KAF for stock price prediction. Thus, this study aims to predict stock movements in an online manner. Although the issue of financial series forecasting is challenging, the goal of this article however is to take one more step towards addressing the issue and to try and lay the groundwork for future work. To do this, we use the National Stock Exchange (NSE), Nifty-50 dataset, which contains 50 leading stocks. In order to describe the contribution of this paper, the following points summarize the essence of the article in brief:

- We propose the use of online-KAF techniques for stock price prediction.
- The data is collected at multiple time windows, i.e., one day, sixty minutes, thirty minutes, twenty five minutes, twenty minutes, fifteen minutes, ten minutes, five minutes, and one minute. The proposed idea is applied to each of these time windows to try and find the best window for stock price prediction.
- The main objective is to predict the closing price of a stock. To do that, we apply ten different KAF-based algorithms and present a comprehensive discussion detailing every aspect of the analysis. With numerical testing performed on all fifty stocks of the main index (Nifty-50), we show the work's efficacy in this article.
- We experiment with two different years. First, we try to predict stock prices for the year 2020. Second, we apply the same set of parameters on the most recent data (2021) and try to show the efficacy of the work. Through experiments performed on these two different years, we have found the method proposed in the paper outperforms similar methods in the literature.
- Lastly, we also try to show that although the KAF class of algorithms is new in the arena of stock prediction, they nevertheless are a practically viable candidate.

The rest of the paper is organized as follows: In Section 2, we discuss the related work. A discussion on different KAF algorithms is presented in Section 3. The experimental results are described in Section 4. Finally, the conclusion is given in Section 5.

## 2. Related Work

Stock price prediction is one of the nontrivial problems of the literature. Numerous studies have attempted to explain that stock price prediction is difficult to implement because of inherent nonstationarity in the data [28, 29]. Previous research has shown that stock market prediction is noisy and chaotic and follows nonlinearity [4, 6, 30]. Nonlinear modelling methodologies have been proven to be effective in modelling systems in a variety of domains such as in [31, 32]. Various applications found different modelling approaches to solve nonlinearity problems such as in [33–35]. In traditional prediction, the techniques were based on technical analysis with standards of resistance, support, and indicators using past prices [36]. Previous research has also studied various linear techniques such as moving averages, autoregressive models, discriminating analyses, and correlations [37, 38]. Much of the current literature on stock market prediction pays particular attention to machine learning (ML) techniques. ML has emerged as another popular area for time series prediction. Among the available popular techniques, machine learning methods are researched mostly due to their capabilities for recognizing complex pattern in stock prices [39–42].

Based on the time-varying and nonlinearity aspect of time series, there is a massive demand for online prediction algorithms. It follows the idea of sequential calculation and generates faster and accurate outcomes [26]. To date, various methods have been developed, such as neural network (NN), kernel adaptive filter (KAF) algorithms, and online support vector regression (SVR) [22]. However, neural networks suffer from slow convergence and significant computing needs. In addition, SVR and kernel methods have no problem of falling into the local optimum. SVR has a strong generalization ability [43], but it is just suitable for smaller data. In addition, a multifilter neural network (MFNN) is also used to predict stock price movement. The performance of the MFNN was found to be better than other NN approaches, SVM, and random forests [44]. In [45], the authors combined support vector machines for regression (SVR) and kernel principal component analysis (KPCA) to enhance prediction accuracy that may help investors for short-term decisions. However, the high dimension of input variables makes the learning process long, and the final model computational complexity becomes very large. These machine learning methods had a drawback of large time consumption during the learning process.

To reduce the computational burden, kernel-based online learning algorithms have become gradually popular [44, 46]. In this respect, recurrent kernel online learning is applied to predict the transaction price of specific products. It was observed that the model was stable with a low dependency to parameter settings [47]. Similarly, convolutional neural networks (CNNs) are also suggested for predicting the next-day prices [48]. In all, there is sufficient literature that suggests that modelling the movement of a stock price is nontrivial. In this respect, adaptive filtering has proved to be a standard option for prediction model for streaming data with nonstationary properties [49–51]. KAF can therefore be used for sequential prediction of stock prices by exploiting the market interdependence. KAF are preferred because they are nonparametric, have low computational complexity, and converge very fast [21, 52–55]. In this domain, multiple algorithms are proposed for nonstationary data. They are preferred due to insensitivity towards design parameters [49]. Multistep predictions for stocks using meta-cognitive recurrent kernel online learning is proposed in [56]. The advantage of the KAF method is that it solves various problems in balancing efficiency and prediction accuracy.

Currently, the use of KAF approaches in the stock price prediction is limited [19, 20]. In [19], a multiple-kernel learning method was proposed to address KAF's two main issues: kernel size and step size. In [20], the idea of the local models was proposed to learn the behavior from different stock markets and compare with other online learning methods such as LSTM, quantized kernel least mean square (QKLMS), nearest instance centroid estimation (NICE), vector autoregression (VAR), and vector error-correction model (VECM) for daily closing price prediction. In another research, the study in [57] proposed the idea of adaptive stock trading strategies with deep reinforcement learning methods focused on extracting informative financial features via two methods: gated deep Q-learning trading strategy (GDQN) and gated deterministic policy gradient trading strategy (GDPG). This paper proposes an online KAF-based learning approach. The selection of basis functions can be done during sample-by-sample training in online kernel learning, which is a more efficient option. This method can be incredibly efficient and successful because they only require one pass over the training data.

To the best of our knowledge, the work presented in this article is the first wherein we comprehensively analyze the price of a stock on multiple time windows and comprehensively test the application of the KAF class of algorithms in stocks. Consequently, to discuss the novel contribution, the following points summarize the fundamental differences between this paper and existing work:

- To the best of our knowledge, we are the first to use KAF algorithms with multiple time windows to analyze and predict stock prices.
- Stock prediction using existing online methods requires a lot of computation time. The article aims to present a general framework wherein the price prediction can be made in a significantly less amount of time.
- Stock traders can quickly sell and buy specific stocks with numerous time windows using the proposed strategy, resulting in larger earnings.

## 3. Methodology

As discussed in Section 1, we have worked with KAF-based techniques. Furthermore, we use online prediction methods. In this regard, KAFs work by self-tuning, where the input-output mapping is formulated according to an optimization criterion usually determined by the error signal. There are two types of adaptive filters: linear and nonlinear. In linear filters, the traditional system follows a supervised technique and depends upon error-correction learning. The filter adaptively adjusts weights, *ω*(*i* − 1), where *i* denotes the discrete-time interval. Here, the input signal *v*_*i*_ is mapped to an actual response *t*_*i*_. Correspondingly, an error is denoted by *e*_*i*_. The error signal adjusts weights by incremental value denoted by Δ*ω*_*i*_. At the next iteration, *ω*_*i*_ becomes the current value of the weight to be updated. This process is continuously repeated until the filter reaches convergence; this generally occurs when the weight adjustment is small enough. Linear adaptive filters do not give satisfactory performance for the nonlinear system due to the results varied in a nonintuitive manner. In real-world problems, where data patterns are more complex, classes may not be separated easily by hyperplanes. Consequently, we have to look to nonlinear methods. In this paradigm, data is projected into high-dimensional linear feature space and prediction is done in this high-dimensional space. Comparing with other existing techniques for regression and classification, KAF has the following advantages:

- KAFs are universal approximators.
- KAFs handle the complexity issues in terms of computation and memory. Moreover, they follow the property of no local minima.
- KAFs follow the idea of online learning and handle nonstationary conditions well.

It was discussed that nonlinear adaptive techniques are well suited for real-world problems. In this regard, kernel methods transform data into a set of points in the RKHS (Reproducing Kernel Hilbert Space). The main idea of KAF can be summarized as the transformation of input data into a high-dimensional feature space *G*, via Mercer kernel. For this, the problem can be solved via inner products. There is no need to do expensive computations in high-dimensional space, owing to the famous “kernel trick.” Considering KAF, suppose we have an input-output mapping as *g* : *𝒱*⟶*R*, based on a well-known sequence ((*v*_1_, *t*_1_), (*v*_2_, *t*_2_),…, (*v*_*i*_, *t*_*i*_)). Here, *v*_*i*_ is the system input with *i* = 1,…, *n* and *t*_*i*_ is equivalent to desired response. The goal is to estimate *g* from data. In KAFs, generally, the computation involves the use of a kernel. An example of a kernel is given as follows:

(1) $$\begin{array}{c} \kappa \langle v,v^{\prime }\rangle =\exp\frac{\left({\left\|v-v^{\prime }\right\|}^{2}\right)}{\sigma^{2}}. \end{array}$$

Here, *κ* denotes the kernel and *σ* denotes the kernel width.

### 3.1. Discussion on KAF Algorithms

In this section, we discuss some of the most popular methods in KAF. For reasons of brevity, we keep the discussion short.

#### 3.1.1. Least Mean Square (LMS) Algorithm

According to [46], the main aim of LMS algorithm is to minimize the following empirical risk function:

(2) $$\begin{array}{c} \underset{\omega }{\min}R_{\text{emp}}\left[\omega \in H_{1},R^{L}\right]=\sum_{i=1}^{N}{\left(t_{i}-\omega \left(v_{i}\right)\right)}^{2}. \end{array}$$

Applying stochastic gradient descent (SGD), equation (2) can be represented as

(3) $$\begin{array}{c} \omega_{0}=0,\\ e_{i}=t_{i}-\omega_{\left(i-1\right)}v_{i},\\ \omega_{i}=\omega_{\left(i-1\right)}+\eta e_{i}v_{i}, \end{array}$$

where *η* is step size and *e*_*i*_ is known as prior error.

The weight-update equation results in the following form:

(4) $$\begin{array}{c} \omega_{i}=\eta \sum_{i=1}^{N}e_{i}v_{i}. \end{array}$$

Representing the idea in terms of inner product, we get

(5) $$\begin{array}{c} t=\omega_{i}\left(v\right)=\eta \sum_{i=1}^{n}e_{i}\langle v_{i},v\rangle ,\\ e_{i}=t_{i}-\eta \sum_{i=1}^{n-1}e_{i}\langle v_{i},v\rangle . \end{array}$$

#### 3.1.2. Kernel Least Mean Square Algorithm (KLMS)

KLMS [21] is an extension of LMS algorithm, the main difference is input *v*_*i*_ is transformed to Ψ(*v*_*i*_) in the high-dimensional space RKHS. Applying LMS algorithm at new sequences {Ψ(*i*), *t*_*i*_}, we get

(6) $$\begin{array}{c} \omega_{0}=0,\\ e_{i}=t_{i}-\omega_{\left(i-1\right)}^{T}\Psi \left(i\right),\\ \omega_{i}=\omega_{\left(i-1\right)}+\eta e_{i}\Psi \left(i\right), \end{array}$$

where *e*_*i*_ is the prediction error, *ω*_*i*_ is the weight vector in *G*, and *η* is the step size.

Using the kernel trick, KLMS can now be written as

(7) $$\begin{array}{c} g_{0}=0,\\ e_{i}=t_{i}-g_{i-1}\left(v_{i}\right),\\ g_{i}=g_{i-1}+\eta e_{i}\kappa \langle v_{i},.\rangle . \end{array}$$

KLMS assigns new unit for every input *v*_*i*_ as the center with *ηe*_*i*_ as its coefficient. Following the radial basis function (RBF), the algorithms are represented as follows:

(8) $$\begin{array}{c} g_{i}=\sum_{j=1}^{i}b_{j}\left(i\right)\kappa \langle v_{j},.\rangle . \end{array}$$

#### 3.1.3. Kernel Recursive Least Square Algorithm (KRLS)

According to [21], in KRLS, the objective function is complemented via a regularization parameter. This can be represented as follows:

(9) $$\begin{array}{c} \underset{\omega }{\min}\sum_{j=1}^{i}{\left|t\left(j\right)-\omega^{T}\Psi \left(j\right)\right|}^{2}+\Lambda {\left\|\omega \right\|}^{2}, \end{array}$$

where Λ stands for regularization vector.

It is shown that *ω*_*i*_=*ψ*(*i*)*b*(*i*), where *b*(*i*)=[Λ*I*+*L*(*i*)]^−1^*t*_*i*_; also, *t*_*i*_=[*t*_1_, *t*_2_, *t*_3_ … .,*t*_*i*_]^*T*^, *L*(*i*)=*ψ*(*i*)^*T*^*ψ*(*i*), and *ψ*(*i*)=[Ψ(1), Ψ(2), Ψ(3),…, Ψ(*i*)].

Complementing the previous equation with RBF, we get

(10) $$\begin{array}{c} g_{i}=\sum_{j=1}^{i}b_{j}\left(i\right)\kappa \langle v_{j},.\rangle . \end{array}$$

The whole idea here can now be summarized as

(11) $$\begin{array}{c} R\left(i-1\right)={\left(\Lambda I+L\left(i-1\right)\right)}^{-1},\\ O\left(i\right)=\psi {\left(i-1\right)}^{T}\Psi \left(i\right),\\ E\left(i\right)=R\left(i-1\right)O\left(i\right),\\ U\left(i\right)=\Lambda +\kappa \langle v_{i},v_{i}\rangle -E{\left(i\right)}^{T}O\left(i\right). \end{array}$$

Following the sequential property of KRLS, we have

(12) $$\begin{array}{c} g_{0}=0,\\ e_{i}=t_{i}-g_{i-1}\left(v_{i}\right),\\ g_{i}=g_{i-1}+U{\left(i\right)}^{-1}e_{i}\kappa \langle v_{i},.\rangle -\sum_{j=1}^{i-1}U{\left(i\right)}^{-1}e_{i}E_{j}\left(i\right)\kappa \langle v_{j},.\rangle . \end{array}$$

KRLS updates all previous coefficients through −*U*(*i*)^−1^*e*_*i*_ *E*(*i*), whereas KLMS never updates previous coefficients. Here, *E*_*j*_(*i*) is the *j*^th^ component of *E*(*i*). The computational complexity of KRLS is *O*(*i*^2^).

#### 3.1.4. Kernel Affine Projection Algorithms (KAPAs)

KAPA [58] derives the idea of KLMS while reducing boosting performance and gradient noise. In KAPA, we formulate with sequences {*t*_1_, *t*_2_} and {Ψ(1), Ψ(2)} to minimize the cost function and estimate with weight vector *ω*.

(13) $$\begin{array}{c} \underset{\omega }{\min}\text{emp}{\left|t-\omega^{T}\Psi \left(v\right)\right|}^{2}. \end{array}$$

Using stochastic gradient descent, we replace covariance matrix and cross covariance vector by local approximation directly from the data. Hence, we get the following equations:

(14) $$\begin{array}{c} \omega_{i}=\omega_{\left(i-1\right)}+\eta \psi \left(i\right)\left[t\left(i\right)-\psi {\left(i\right)}^{T}\omega_{\left(i-1\right)}\right], \end{array}$$

where *ψ*(*i*)=[Ψ(*i* − *K*+1),…, Ψ(*i*)] and K is the observation and regressor.

#### 3.1.5. Quantized Kernel Least Mean Square Algorithm (QKLMS)

QKLMS is a famous algorithm proposed in [50]. It is an extension of KLMS algorithm to deal with the issue of data redundancy. Using quantization operator the core idea can be written as

(15) $$\begin{array}{c} \omega_{0}=0,\\ e_{i}=t_{i}-\omega_{\left(i-1\right)}^{T}\Psi \left(i\right),\\ \omega_{i}=\omega_{\left(i-1\right)}+\eta e_{i}Q\left[\Psi \left(i\right)\right], \end{array}$$

where, in feature space *G*, 𝒬[.] denotes the quantization. The learning rule for QKLMS is

(16) $$\begin{array}{c} g_{0}=0,\\ e_{i}=t_{i}-g_{i-1}\left(v_{i}\right),\\ g_{i}=g_{i-1}+\eta e_{i}\kappa \left(Q\left[v_{i}\right],.\right). \end{array}$$

QKLMS and KLMS have almost the same computational complexity. The only difference between the two algorithms is that QKLMS deal with the issue of data redundancy to locally update the coefficients of closest center.

In short, the central theme of QKLMS is given in Algorithm 1.

#### 3.1.6. Kernel Normalized Least Mean Square Algorithm (KNLMS)

According to [49], KNLMS algorithm is used for dictionary designing with coherence criterion. Here, we discuss KNLMS from the point of view of MKNLMS-CS (multikernel normalized least mean square algorithm with coherence based sparsification).

Assume *κ*_*m*_ : *𝒱* × *𝒱*⟶*R*, where *m* ∈ ℳ : {1,2,…, *M*} is a set of *M* distinct kernels.

Consider *𝒥*_*n*_^*cs*^≔*j*_1_^(*n*)^, *j*_2_^(*i* − 1)^,…, *j*_*r*_*n*__^(*i* − 1)^ ⊂ {0,1,…, *n* − 1} to be the dictionary {*κ*_*m*_(.,*v*_*j*_)}_*m*∈ℳ_*j*∈*𝒥*_*n*_^*cs*^__.

Here, *r*_*n*_≔|*𝒥*_*n*_^*cs*^| is the size of dictionary. The filter works as per the following set of rules:

(17) $$\begin{array}{c} \Psi_{n}^{cs}\left(v\right)=\sum_{m\in ℳ}\sum_{j\in J_{n}^{cs}}h_{j,n}^{\left(m\right)}\kappa_{m}\left(v,v_{j}\right),\;v\in V, \end{array}$$

where *h*_*j*,*n*_^(*m*)^ ∈ *R*, *m* ∈ ℳ, *j* ∈ *𝒥*_*n*_^*cs*^. The estimated error ${\hat{t}}_{n}≔\Psi_{n}^{cs}\left(v_{n}\right)$ of *t*_*n*_ can be written as

(18) $$\begin{array}{c} \Psi_{n}^{cs}\left(v_{n}\right)=\sum_{j\in J_{n}^{cs}}h_{j,n}^{T}\kappa_{j,n}, \end{array}$$

where

(19) $$\begin{array}{c} \kappa_{j,n}≔{\left[\kappa_{1}\left(v_{n},v_{j}\right),\kappa_{2}\left(v_{n},v_{j}\right),\ldots ,\kappa_{M}\left(v_{n},v_{j}\right)\right]}^{T}\in R^{M},\\ h_{j,n}≔{\left[h_{j,n}^{\left(1\right)},h_{j,n}^{\left(2\right)},h_{j,n}^{\left(3\right)},\ldots ,h_{j,n}^{\left(M\right)}\right]}^{T}\in R^{M}. \end{array}$$

Let the initial dictionary be indicated as *𝒥*_0_^*cs*^≔∅. This makes *H*_0_ to be an empty size matrix. Following algorithm, we only add a new point *n* into *𝒥*_*n*_^*cs*^ if the following condition holds:

(20) $$\begin{array}{c} {\left\|\kappa \right\|}_{\max}≔\underset{m\in ℳ}{\max}\underset{j\in J_{n}^{cs}}{\max}\left|\kappa_{m}\left(v_{n},v_{j}\right)\right|\leq \Delta ,\;n\in N, \end{array}$$

where Δ > 0 is the threshold. Let *η* ∈ [0,2] denote the step size and Λ > 0 denote the regularization parameter. The update rule is given as follows:

(i) If equation (20) is satisfied, *𝒥*_*n*+1_^*cs*^≔*𝒥*_*n*_^*cs*^ ∪ {*n*}. Also,
  (21) $$\begin{array}{c} H_{n+1}≔{\bar{H}}_{n}+\eta \frac{t_{n}-\langle {\bar{K}}_{n},{\bar{H}}_{n}\rangle }{{\left\|{\bar{K}}_{n}\right\|}^{2}+\Lambda }{\bar{K}}_{n}. \end{array}$$
(ii) If equation (20) is not satisfied, *j*_*n*+1_^*cs*^≔*j*_*n*_^*cs*^. Also,
  (22) $$\begin{array}{c} H_{n+1}≔H_{n}+\eta \frac{t_{n}-\langle H_{n},K_{n}\rangle }{{\left\|K_{n}\right\|}^{2}+\Lambda }K_{n}, \end{array}$$

where ${\bar{H}}_{n}≔\left[\begin{array}{cc} H_{n} & 0 \end{array}\right]$ and ${\bar{K}}_{n}≔\left[\begin{array}{cc} K_{n} & {\bar{k}}_{n} \end{array}\right]$ with ${\bar{k}}_{n}≔{\left[\kappa_{1}\left(v_{n},v_{n}\right),\kappa_{2}\left(v_{n},v_{n}\right),\kappa_{3}\left(v_{n},v_{n}\right),\ldots ,\kappa_{M}\left(v_{n},v_{n}\right)\right]}^{T}$ where 0 ∈ *R*^*M*^ is the zero vector. For KNLMS, the value of *M* is 1.

#### 3.1.7. Probabilistic Least Mean Square Algorithm (PROB-LMS)

The probabilistic approach to the LMS filter is an efficient approximation method. It provides an adaptable step-size LMS algorithm together with a measure of uncertainty about the estimation. In addition, it also preserves the linear complexity of the standard LMS. Some of the advantages of probabilistic models are as follows: (1) they force the designer to specify all the assumptions of the model, (2) they provide a clear separation between the model and the algorithm used to solve it, and (3) they usually provide some measure of uncertainty about the estimation. It is assumed observation models to be Gaussian with this distribution:

(23) $$\begin{array}{c} pr\left(t_{k}|\omega_{k}\right)=N\left(t_{k};v_{k}^{T}\omega_{k},\sigma_{n}^{2}\right), \end{array}$$

where *ω*_*k*_ = parameter vector, *σ*_*n*_^2^ = variance for observation noise, and *v*_*k*_ = regression vector.

#### 3.1.8. Kernel Maximum Crossentropy Criterion (KMCC)

The algorithm's main aim is to maximize crossentropy between desired *t*_*i*_ and actual output *y*_*i*_ [55]. Using MCC criterion and SGD, the algorithm can be written as

(24) $$\begin{array}{c} \omega_{0}=0,\\ \omega_{\left(i+1\right)}=\omega_{i}+\eta \frac{\partial \kappa_{\sigma }\left(t_{i},\omega_{i}^{T}\Psi \left(v_{i}\right)\right)}{\partial \omega_{i}}\\ =\omega_{i}+\eta \left[\left(\exp\frac{\left(-e_{i}^{2}\right)}{2\sigma^{2}}\right)e_{i}\Psi \left(i\right)\right]\ldots \\ =\eta \sum_{i=1}^{n}\left[\left(\exp\frac{\left(-e_{i}^{2}\right)}{2\sigma^{2}}\right)e_{i}\Psi \left(i\right)\right], \end{array}$$

where *σ* is the kernel width and *η* is the step size.

The complete prediction and error calculation can be summarized as

(25) $$\begin{array}{c} y_{i}=\eta \sum_{i=1}^{n}\left[\left(\exp\frac{\left(-e^{2}\right)}{2\sigma^{2}}\right)e_{i}\kappa \langle v_{i},v_{n}\rangle \right],\\ e_{i}=t_{i}-y_{i}. \end{array}$$

#### 3.1.9. Leaky Kernel Affine Projection Algorithm (LKAPA)

The LKAPA [58] is the extension of KAPA discussed in Section 3.1.4. According to equation (14), weight updation is a difficult task in high-dimensional space. Here equation (14) is modified. This can be done as follows:

(26) $$\begin{array}{c} \omega_{0}=0,\\ \psi {\left(i\right)}^{T}\omega_{\left(i-1\right)}={\left[\sum_{j=1}^{i-1}b_{j}\left(i-1\right)\kappa_{i-K+1,j},\ldots ,\sum_{j=1}^{i-1}b_{j}\left(i-1\right)\kappa_{i-1,j},\sum_{j=1}^{i-1}b_{j}\left(i-1\right)\kappa_{i,j}\right]}^{T},\\ e_{i}=t_{i}-\psi {\left(i\right)}^{T}\omega_{\left(i-1\right)},\\ \omega_{i}=\omega_{\left(i-1\right)}+\eta \psi \left(i\right)e_{i}\\ =\sum_{j=1}^{i=1}b_{j}\left(i-1\right)\Psi \left(j\right)+\sum_{j=1}^{K}\eta e_{j}\left(i\right)\Psi \left(i-j+K\right), \end{array}$$

where *κ*_*i*,*j*_=*κ*(*v*(*i*), *v*(*j*))

The weight vector is computed using the following criterion:

(27) $$\begin{array}{c} \omega_{i}=\sum_{j=1}^{i}b_{j}\left(i\right)\Psi \left(i\right),\;\forall_{i}\geq 0. \end{array}$$

From the perspective of empirical risk minimization, we minimize the following objective function:

(28) $$\begin{array}{c} \underset{\omega }{\min}\text{emp}{\left|t-\omega^{T}\Psi \left(v\right)\right|}^{2}+\Lambda {\left\|\omega \right\|}^{2}. \end{array}$$

Then, we get

(29) $$\begin{array}{c} \omega_{i}=\left(1-\Lambda \eta \right)\omega_{\left(i-1\right)}+\eta \psi \left(i\right)\left[t\left(i\right)-\psi {\left(i\right)}^{T}\omega_{\left(i-1\right)}\right], \end{array}$$

where *ψ*(*i*)=[Ψ(*i* − *K*+1),…, Ψ(*i*)]

Finally, coefficient *b*_*κ*_(*i*) is updated as

(30) $$\begin{array}{c} b_{\kappa }\left(i\right)=\left\{\begin{array}{c} \eta \left(t_{i}-\sum_{j=1}^{i-1}b_{j}\left(i-1\right)k_{i,j}\right.,\;k=i,\\ \text{for}\left\{i-K+1\leq k\leq i-1\right\},\\ \begin{array}{c} \left(1-\Lambda \eta \right)b_{k}\left(i-1\right)+\eta \left(t\left(k\right)-\sum_{j=1}^{i-1}b_{j}\left(i-1\right)\kappa_{k,j}\right),\\ 1\leq k<i-K+1,\left(1-\Lambda \eta \right)b_{k}\left(i-1\right). \end{array} \end{array}\right. \end{array}$$

#### 3.1.10. Normalized Online Regularized Risk Minimization Algorithm (NORMA)

NORMA [58] is one of the kernel-based version of LKAPA described in Section 3.1.9. It is also correlated with the KLMS algorithm summarized in Section 3.1.2. NORMA includes the regularization and nonlinear functional approach. It allows to reject old values ones in a sliding window manner.

### 3.2. Problem Formulation

In this section, we discuss the results of stock prediction using all the ten discussed algorithms. The purpose of stock prediction is to determine the future values of a stock depending upon the historical values. As discussed in the Introduction section, our main aim is to predict the close price. To this end, we calculated the percentage change in close price. Subsequently, we apply the idea of autoregression of the order *m* to predict the future change in the stock price. An autoregressive (AR) model forecasts future behavior using data from the past. When there is a correlation between the values in a time series and the values that precede and succeed them. In such situations, AR models have shown tremendous potential. In context of the work presented here, the problem is formulated as

(31) $$\begin{array}{c} \Psi \left(V_{i}\right)=\sum_{i=1}^{m}\omega_{\left(i-1\right)}\Psi \left(V_{i}\right). \end{array}$$

**Figure 1:**
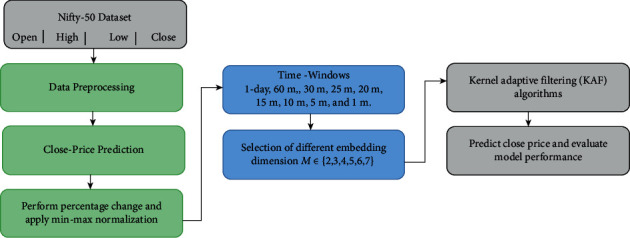
Proposed close price prediction framework.

Ψ(*V*_*i*_) is the close price in the high-dimensional space, and *ω* is the weight vector. Since we follow the AR model, it is imperative to estimate the weight vector. To estimate the weight vector, KAF techniques discussed in the previous section are used. A sample of the formulation is shown in Table 1. In this table, we have shown problem formulation by considering the daywise closing price. This type of procedure is followed commonly in multivariate time series prediction, e.g., [59, 60]. It should be noted here that the procedure was followed for all the window sizes. Subsequently, the problem became autoregression-based next percentage prediction. The actual closing can then be computed from the percentage change easily. The overall framework followed in the article is shown in Figure 1. The experiments were performed on the Nifty-50 dataset, and the data used in the experimentation is available at shorturl.at/lnvF2. The kernel adaptive filtering (KAF) algorithms that were used in this work is available at https://github.com/steven2358/kafbox.

## 4. Experimental Results

### 4.1. Dataset Description

In this section, we have described the experimental details of the Nifty-50 dataset. Nifty-50 is the largest stock exchange in India according to the rate of total and average daily turnover for equity shares. We collected the data of all stocks from 9 : 15 to 3 : 30. In addition, we collected the data for two different periods of years. First, we try to predict stock prices for the year 2020 from January 01, 2020, to December 31, 2020, and second, from the most recent data (2021) between January 01, 2021, and May 31, 2021. The original data was available for one-minute open, high, low, and close (OHLC) prices. From this granular data, we clubbed the OHLC quotes to get the data for other time windows. In particular, we created and preprocessed the dataset according to nine prediction windows (one day, sixty minutes, thirty minutes, twenty five minutes, twenty minutes, fifteen minutes, ten minutes, five minutes, and one minute). Recall that we focused on predicting the percentage changes in close price. To that end, we also normalized the data between the range of 0 to 1. Then, ten distinct KAF algorithms were applied to the final preprocessed data for every stock. Finally, it is worth noting that the experimental findings obtained with the KAF algorithm on the Nifty-50 dataset demonstrate the work's superiority and could serve as a new benchmark for the field's future state of the art.

### 4.2. Evaluation Criterion

To evaluate and compare the performance of various KAF algorithms, we use standard error evaluation metrics such as mean squared error (MSE), mean absolute error (MAE), and directional symmetry (DS). The metrics are elaborated in the following text.

#### 4.2.1. Minimum Square Error (MSE)

MSE is also known as mean squared deviation (MSD) which calculates the average squared difference between the actual and predicted observation:

(32) $$\begin{array}{c} MSE=\sum_{i=1}^{n}{\left(a_{i}-p_{i}\right)}^{2}. \end{array}$$

#### 4.2.2. Mean Absolute Error (MAE)

MAE calculates the average magnitude between actual and predicted observations in a set of predictions, without observing their directions, i.e., the average prediction error.

(33) $$\begin{array}{c} MAE=\frac{1}{n}\sum_{i=1}^{n}\left|a_{i}-p_{i}\right|. \end{array}$$

#### 4.2.3. Directional Symmetry (DS)

Directional symmetry in terms of time series analysis measures the model's performance to predict positive and negative trends from one time period to the next.

(34) $$\begin{array}{c} DS=\frac{1}{n}\sum_{i=1}^{n}d_{i}, \end{array}$$

where

(35) $$\begin{array}{c} d_{i}=\left\{\begin{array}{cc} 0, & \text{otherwise,}\\ 1, & \left(a_{i}-a_{i-1}\right)\left(p_{i}-p_{i-1}\right)\geq 0, \end{array}\right. \end{array}$$

where *n* is the time-step, *a*_*i*_ represents the actual values, and *p*_*i*_ represents the predicted output. In the following procedure, we discuss the details to compute error values.

### 4.3. Procedure: Error Computation

1. We worked with Nifty-50 firms with 2020 and 2021 datasets, as mentioned in Section 1. Moreover, it was also pointed out that we work with ten different algorithms. The parameters listed in Table 2 were tuned manually. In order to find the optimal values of the parameters, multiple experiments were performed.
2. To compute the error values for each stock and every algorithm, we formulated the problem as an autoregressive problem (see Section 3.2) and computed the error values for all 50 stocks. In total, we get 50X3 error values, one for MSE, MAE, and DS. Moreover, we pointed out that we have nine different prediction windows. Hence, error estimation was done for all stocks, all windows, and all ten algorithms.
3. Subsequently, for a particular algorithm, and for a single time window, we take the average of all 50-error metrics (one for every stock) to come up with the final number. The number is presented in the article. This number shows the overall predictive capability of the model on all fifty stocks.

### 4.4. Prediction, Convergence, and Residual Analysis

**Figure 2:**
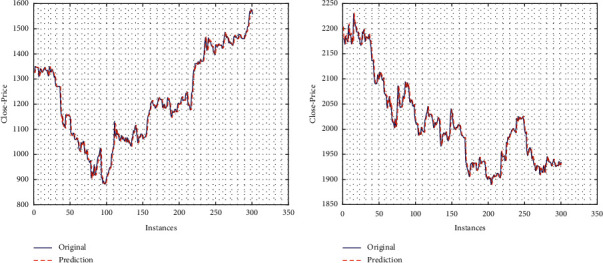
Prediction for one stock (Reliance) using KRLS: (a) 2020 dataset and (b) 2021 dataset.

In this section, we analyze the performance of KAF algorithms for close price prediction. In this regard, the prediction graph for one stock (Reliance) with KRLS is considered.  Figure 2 shows the results for 2020 and 2021 datasets. It is visible from the figure that we are getting good results. It should be noted here that we have presented the result for one stock (Reliance) and one prediction window (sixty minutes). Similar results were obtained for other companies in the dataset. It is also visible from the figure that although the prediction is not cent percent accurate, it is close. It, therefore, implies the superior performance of KAF algorithms in prediction. It should be noted here that although we are getting good results, there are always chances of overfitting. In this article, since we are using online learning, the architecture itself naturally minimizes the chances of overfitting, but it is possible that the superior results might be due to overfitting.

**Figure 3:**
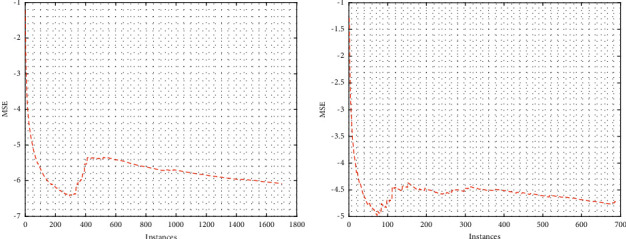
Error convergence for one stock (Reliance) using KRLS: (a) 2020 dataset and (b) 2021 dataset.

It is expected from any machine learning algorithm that it should converge as we train the model with more instances; in other words, the error as we progress through the training should decrease to an acceptable range. In this regard and in addition to presenting the results for prediction in Figure 2, we have also presented the result of convergence in Figure 3 for 2020 and 2021 datasets, respectively. Similar to the previous case, we have only plotted the result considering single stock (Reliance) and one prediction time window. The convergence graphs of the algorithm were plotted taking MSE as the error metrics. Figure 3 shows the error convergence graph for both the datasets and KRLS algorithm for the Reliance stock. In Figure 3, *x*-axis shows the number of instances and *y*-axis shows the MSE. It can be seen from Figure 3 that the algorithm reached convergence very quickly. In fact, the algorithm reached convergence at 1000th data point. Convergence is very important in KAF as it shows the ability of the algorithm to adapt itself and learn from the data quickly. Though, there are minor fluctuations in the end, but it nevertheless is acceptable as there will always be minor changes in the new data.

**Figure 4:**
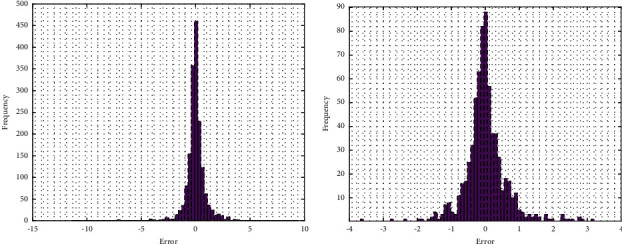
Error residuals for one stock (Reliance) using KRLS: (a) 2020 dataset and (b) 2021 dataset.

To complement the prediction results, we have also presented the distribution of error residuals in Figure 4 for 2020 and 2021 datasets, respectively. As visible from the figure, the residuals are following a normal distribution. This type of behavior is excellent as there are very few outliers. Moreover, the overall variance of the residuals is also less, showing the excellent prediction potential of the algorithm.

### 4.5. Comprehensive Evaluation of KAF Algorithms

In contrast to batch learning techniques, which generate the best predictor by learning on the full training dataset at once, we employ an online learning concept in which data becomes available in a sequential order (sample by sample training) and is used to update the best predictor for future data at each step. As we have used ten different algorithms, it is logical to compare the performance of all algorithms. In this regard, we have shown the result in two different datasets. First, we attempt to forecast stock prices for 2020. Second, we use the same set of parameters on the most recent data (2021) to demonstrate the work's efficacy. To evaluate the performance of KAF-based methods, we try to experiment with different values of *M* (the embedding dimension). We vary the underlying dimensions from 2 to 7 with a step size of 1, i.e., *M* ∈ {2,3,4,5,6,7}. With this setup, the results are presented in Tables 3 and 4. It is visible from the table that once again, KRLS performed well in terms of error minimization. The best number for the embedding dimension is two when we consider MSE and MAE. However, when it came to DS, the numbers and the algorithms are different because a market trend is a term used to describe how a market moves over time. A trend can generally move upward or downward. For instance, considering daily data (1 day in the table), the best performing algorithm is LKAPA with embedding dimension (*M*)=5. In fact, for this metric (DS), we see much conflict in terms of the best algorithm. Nevertheless, the experimentation revealed the superiority of KRLS, PROB-LMS, and LKAPA.

### 4.6. Comparison with Other State-of-the-Art Methods

We compared our result with other learning methods such as [61–63], among other learning approaches. The deep learning (DL) algorithms were taught and assessed over a period of 25 epochs utilizing an 80 : 20 split. The amount of time taken to train and make prediction was recorded. Based on the architecture details and hyperparameter settings provided in the relevant articles, the DL-based method [61–63] stocks were reimplemented. All of the techniques were trained on the Nifty-50 dataset. We chose fifty equities for the sixty-minute time periods to maintain uniformity across different ways for experimentation. In terms of MSE, RMSE, and execution time, all of the approaches were then compared to the suggested KAF method (KRLS). For the 2020 and 2021 datasets, Tables 5 and 6 summarize the comparative outcomes learning approaches. The results in Tables 5 and 6 show that the proposed approach outperforms previous stock prediction methods in the literature.

We must point it out here that since all the models belong to the same category of kernel adaptive filtering, the complexity of all the models is almost similar. For neural networks used in the article, we collect the architecture from their respective papers [61–63]. It should be noted here that KAF is also analogous to the neural network architecture with a single layer. Furthermore, even though it has a single layer, it is giving good results.

### 4.7. Experimentation with Dictionary Size

In addition to the experiment conducted in the previous section, we have also experimented with the dictionary size of KAF algorithms. The result for this experiment is presented in Table 7. As visible, increasing the dictionary size decreases the performance of the system. Moreover, increasing the dictionary size also increased the execution time. It should be noted here that the execution time for predicting the next closing price for a single stock with dictionary size 500 is 0.675 seconds. This figure (0.675 seconds) clearly shows the applicability of the KAF class of algorithms in high-frequency trading, where latency is a key factor.

### 4.8. Important Note: Error Minimization and Profitability

From Tables 3 and 4, we can see that KRLS performed well in minimizing error. Moreover, the lowest error (MSE) that we get is in the order of 10^−4^. It should be noted here that we got this error for the time window of one minute. In this regard, it is common sense that if we minimize the error, we can get close to the actual values, which is indeed true. However, considering the time window of one minute, there is an issue. In this interval, the fluctuation in the price is low. This means that minimizing error will not result in too much profit. In other words, the volatility in one minute is less. Hence, predictions are very close. However, the chances of taking a position and getting profit in a low volatile environment is also very less. Therefore, one has to maintain a balance between error minimization and profitability.

## 5. Conclusion

This paper introduces a framework to predict stock prices using KAF. We comprehensively analyzed the Indian Financial Sector, Nifty-50, and showed the predictive results of all 50 stocks in the main index. We experimented with ten different algorithms belonging to the KAF class of algorithms. Experimentation was performed on nine different windows starting at one minute and progressing to one day. This is the first time, to our knowledge, that numerous KAF algorithms have been implemented at such granular levels. The evidence offered in the Experimental Results section demonstrated the work's overall predictive capability. It was discovered that the KAF class of algorithms not only outperformed other algorithms in terms of error minimization but also had a very short execution time, underlining its usefulness in the field of high-frequency trading.

For future work, we would test the framework via the application of hyperparameter optimization. This would be beneficial because KAF algorithms must deal with a wide range of hyperparameter settings. We will also use several hyperparameter optimization strategies to improve the model's accuracy.

## Figures and Tables

**Algorithm 1:**
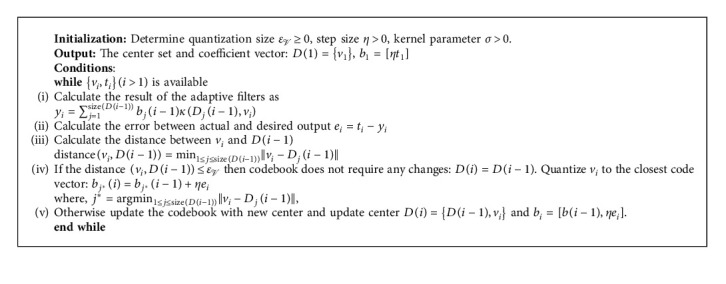
The central idea of the QKLMS algorithm.

**Table 1:** Time window of 1 day (stock-Reliance).

Day	Actual price	Change in price
1 day	1987.5	0.1685
2 day	1990.85	−1.2431
3 day	1966.1	−2.6372
4 day	1914.25	−0.16194
5 day	1911.15	1.17991
6 day	1933.7	−1.8849
7 day	1897.25	3.1519
8 day	1957.05	−0.9325
9 day	1938.8	1.1244
10 day	1960.6	—

If we choose *M* = 3, then **Input** = [{0.1685,-1.2431,-2.6372}] and **Output** = [{−0.16194}].

**Table 2:** Parameter description for close price using ten different KAF algorithms.

Parameter	KAPA	KLMS	KMCC	KNLMS	KRLS	LKAPA	LMS	NORMA	PROB-LMS	QKLMS
(*σ*)	4.0	4.0	4.0	4.0	3.0	5.0		7.0		3.0
(*η*)	1.7	1.1	1.5	1.7		0.09		1.1		1.2
(*ɛ*)	1E-4			1E-2						0.3
(Λ)						1E-4		1E-2	0.4	
(*σ*_2_*n*)									2	
(*σ*_2_*d*)									6	
mu0	0.2			2			0.2			
(P)	20					20				
nu					1E-2					
*τ*								500		
tcoff								0.9		

*σ* = kernel width, *σ*_2_*n* = variance of observation noise, *σ*_2_*d* = variance of filter weight diffusion, *η* = step size, *ɛ* = regularization parameter, Λ = Tikhonov regularization, tcoff = learning rate coefficient, *τ* = memory size (terms retained in truncation), mu0 = coherence criterion threshold, *P* = memory length, and nu = approximate linear dependency (ALD) threshold.

**Table 3:** Best embedding dimension for all time windows according to evaluation metrics MSE, MAE, and DS (2020).

Time window	MSE	Best embedding dimensions	Algorithms

1 day	0.014 3	2	KRLS
60 minutes	0.003 4	2	KRLS
30 minutes	0.002 0	2	KRLS
25 minutes	0.001 8	2	KRLS
20 minutes	0.001 5	2	KRLS
15 minutes	0.001 2	2	KRLS
10 minutes	0.000 8	2	KRLS
5 minutes	0.000 4	2	KRLS
1 minute	0.000 10	2	KRLS
Time window	MAE	Best embedding dimensions	Algorithms
1 day	2.132 4	2	KRLS
60 minutes	0.660 2	2	KRLS
30 minutes	0.466 6	2	KRLS
25 minutes	0.431 7	2	KRLS
20 minutes	0.381 2	2	KRLS
15 minutes	0.329 5	2	KRLS
10 minutes	0.266 7	2	KRLS
5 minutes	0.188 6	2	KRLS
1 minute	0.085 2	2	KRLS
Time window	DS	Best embedding dimensions	Algorithms
1 day	0.501 3	5	LKAPA
60 minutes	0.491 9	5	KRLS
30 minutes	0.491 3	5	KRLS
25 minutes	0.492 5	3	KRLS
20 minutes	0.488 1	2	PROB-LMS
15 minutes	0.489 3	7	QKLMS
10 minutes	0.490 2	2	KRLS
5 minutes	0.491 0	2	PROB-LMS
1 minute	0.471 5	2	KRLS

**Table 4:** Best embedding dimension for all time windows according to evaluation metrics MSE, MAE, and DS (2021).

Time window	MSE	Best embedding dimensions	Algorithms
1 day	0.034 2	2	KRLS
60 minutes	0.008 1	2	KRLS
30 minutes	0.004 7	2	KRLS
25 minutes	0.004 2	2	KRLS
20 minutes	0.003 3	2	KRLS
15 minutes	0.002 8	2	KRLS
10 minutes	0.002 0	2	KRLS
5 minutes	0.001 1	2	KRLS
1 minute	0.000 3	2	KRLS
Time window	MAE	Best embedding dimensions	Algorithms
1 day	1.690 1	2	KRLS
60 minutes	0.548 0	2	KRLS
30 minutes	0.396 1	2	KRLS
25 minutes	0.367 1	2	KRLS
20 minutes	0.323 8	2	KRLS
15 minutes	0.280 3	2	KRLS
10 minutes	0.225 9	2	KRLS
5 minutes	0.160 1	2	KRLS
1 minute	0.072 9	2	KRLS
Time window	DS	Best embedding dimensions	Algorithms
1 day	0.487 0	4	NORMA
60 minutes	0.493 0	4	KNLMS
30 minutes	0.484 9	4	KRLS
25 minutes	0.487 8	6	LKAPA
20 minutes	0.489 1	7	LKAPA
15 minutes	0.488 1	2	PROB-LMS
10 minutes	0.489 1	2	PROB-LMS
5 minutes	0.484 6	2	PROB-LMS
1 minute	0.475 1	2	KRLS

**Table 5:** Comparison of the proposed work with other state-of-the-art stock prediction method for 60-minute time window (2020 dataset) from January 01, 2020, to December 31, 2020.

Method	MSE	RMSE	Execution time (s)
Gao et al. [61]	0.519 17	0.7205	400.39
Moghar et al. [62]	0.518 00	0.7197	1265.11
Nikou et al. [63]	0.518 38	0.7199	5006.19
Proposed method	0.003 4	0.0583	5.234

**Table 6:** Comparison of the proposed work with other state-of-the-art stock prediction method for 60-minute time window (2021 dataset) from January 01, 2021 to May 31, 2021.

Method	MSE	RMSE	Execution time (s)
Gao et al. [61]	0.702 02	0.8378	362.67
Moghar et al. [62]	0.697 5	0.8351	1082.90
Nikou et al. [63]	0.702 32	0.8380	2250.87
Proposed method	0.008 1	0.09	4.256

**Table 7:** Effect of dictionary size.

Dictionary size	MSE	MAE	DS	Execution time (seconds)
500 dictionary size	0.020 2	0.687	0.511	0.675
1000 dictionary size	0.019 3	0.674	0.497	0.696
5000 dictionary size	0.019 3	0.674	0.497 1	0.702

The algorithm chose KMCC (60 minutes, 2021 dataset).

## Data Availability

The datasets and all related materials are available for download from the following website: shorturl.at/lnvF2.

## References

[1] Agrawal A., Gans J., Goldfarb A. (2018). *Prediction Machines: The Simple Economics of Artificial Intelligence*.

[2] Goodwin P., Önkal D., Thomson M. (2010). Do forecasts expressed as prediction intervals improve production planning decisions?. *European Journal of Operational Research*.

[3] Karevan Z., Suykens J. A. K. (2020). Transductive lstm for time-series prediction: an application to weather forecasting. *Neural Networks*.

[4] Abraham A., Philip N. S., Saratchandran P. (2004). Modeling chaotic behavior of stock indices using intelligent paradigms. https://arxiv.org/abs/cs/0405018.

[5] Berger D., Pukthuanthong K., Jimmy Yang J. (2011). International diversification with frontier markets. *Journal of Financial Economics*.

[6] Singh R., Srivastava S. (2017). Stock prediction using deep learning. *Multimedia Tools and Applications*.

[7] Mishra S., Ahmed T., Mishra V. K. Close-price prediction using online kernel adaptive filtering.

[8] Fama E. F. (1970). Efficient capital markets: a review of theory and empirical work. *The Journal of Finance*.

[9] Clements M. P., Franses P. H., Swanson N. R. (2004). Forecasting economic and financial time-series with non-linear models. *International Journal of Forecasting*.

[10] Guresen E., Kayakutlu G., Daim T. U. (2011). Using artificial neural network models in stock market index prediction. *Expert Systems with Applications*.

[11] Liang X., Zhang H., Xiao J., Chen Y. (2009). Improving option price forecasts with neural networks and support vector regressions. *Neurocomputing*.

[12] Chan L. K. C., Lakonishok J., Swaminathan B. (2007). Industry classifications and return comovement. *Financial Analysts Journal*.

[13] Patel J., Shah S., Thakkar P., Kotecha K. (2015). Predicting stock market index using fusion of machine learning techniques. *Expert Systems with Applications*.

[14] Sagala T. W., Saputri M. S., Mahendra R., Budi I. Stock price movement prediction using technical analysis and sentiment analysis.

[15] Moghaddam A. H., Moghaddam M. H., Esfandyari M. (2016). Stock market index prediction using artificial neural network. *Journal of Economics, Finance and Administrative Science*.

[16] Pimenta A., Nametala C. A. L., Guimarães F. G., Carrano E. G. (2018). An automated investing method for stock market based on multiobjective genetic programming. *Computational Economics*.

[17] Huang S.-C., Chiou C.-C., Chiang J.-T., Wu C.-F. (2020). A novel intelligent option price forecasting and trading system by multiple kernel adaptive filters. *Journal of Computational and Applied Mathematics*.

[18] Huang T.-C., Zaeem R. N., Barber K. S. (2019). It is an equal failing to trust everybody and to trust nobody. *ACM Transactions on Internet Technology*.

[19] Garcia-Vega S., Zeng X.-J., Keane J. (2019). Learning from data streams using kernel least-mean-square with multiple kernel-sizes and adaptive step-size. *Neurocomputing*.

[20] Garcia-Vega S., Zeng X.-J., Keane J. (2020). Stock returns prediction using kernel adaptive filtering within a stock market interdependence approach. *Expert Systems with Applications*.

[21] Weifeng Liu W., Il Park I., Principe J. C. (2009). An information theoretic approach of designing sparse kernel adaptive filters. *IEEE Transactions on Neural Networks*.

[22] Han M., Zhang S., Xu M., Qiu T., Wang N. (2018). Multivariate chaotic time series online prediction based on improved kernel recursive least squares algorithm. *IEEE transactions on cybernetics*.

[23] Liu W., Principe J. C., Haykin S. (2011). *Kernel Adaptive Filtering: A Comprehensive Introduction*.

[24] Candela J. Q., Winther O. (2003). Incremental Gaussian processes. *Advances in Neural Information Processing Systems*.

[25] Yu L., Wang S., Lai K. K. (2007). An online learning algorithm with adaptive forgetting factors for feedforward neural networks in financial time series forecasting. *Nonlinear Dynamics and Systems Theory*.

[26] Pardo J., Zamora-Martínez F., Botella-Rocamora P. (2015). Online learning algorithm for time series forecasting suitable for low cost wireless sensor networks nodes. *Sensors*.

[27] Guo T., Xu Z., Yao X., Chen H., Aberer K., Funaya K. Robust online time series prediction with recurrent neural networks.

[28] Zhang N., Lin A., Shang P. (2017). Multidimensionalk-nearest neighbor model based on EEMD for financial time series forecasting. *Physica A: Statistical Mechanics and Its Applications*.

[29] Tay F. E. H., Cao L. (2001). Application of support vector machines in financial time series forecasting. *Omega*.

[30] Bezerra P. C. S., Albuquerque P. H. M. (2017). Volatility forecasting via SVR-GARCH with mixture of Gaussian kernels. *Computational Management Science*.

[31] Freitas L. O., Henriques P. R., Novais P. (2020). Analysis of human activities and identification of uncertain situations in context-aware systems. *International Journal of Artificial Intelligence*.

[32] Precup R.-E., Teban T.-A., Albu A., Borlea A.-B., Zamfirache I. A., Petriu E. M. (2020). Evolving fuzzy models for prosthetic hand myoelectric-based control. *IEEE Transactions on Instrumentation and Measurement*.

[33] Pozna C., Precup R.-E. (2014). Applications of signatures to expert systems modelling. *Acta Polytechnica Hungarica*.

[34] Yuhana U. L., Fanani N. Z., Yuniarno E. M., Rochimah S., Koczy L. T., Purnomo M. H. (2020). Combining fuzzy signature and rough sets approach for predicting the minimum passing level of competency achievement. *International Journal of Artificial Intelligence*.

[35] Hedrea E.-L., Precup R.-E., Roman R.-C., Petriu E. M. (2021). Tensor product-based model transformation approach to tower crane systems modeling. *Asian Journal of Control*.

[36] Chen Y.-S., Cheng C.-H., Tsai W.-L. (2014). Modeling fitting-function-based fuzzy time series patterns for evolving stock index forecasting. *Applied Intelligence*.

[37] Kumar M., Thenmozhi M. (2014). Forecasting stock index returns using arima-svm, arima-ann, and arima-random forest hybrid models. *International Journal of Banking, Accounting and Finance*.

[38] Wang J.-J., Wang J.-Z., Zhang Z.-G., Guo S.-P. (2012). Stock index forecasting based on a hybrid model. *Omega*.

[39] Mauboussin M. J. (2002). Revisiting market efficiency: the stock market as a complex adaptive system. *The Journal of Applied Corporate Finance*.

[40] Fama E. F. (1995). Random walks in stock market prices. *Financial Analysts Journal*.

[41] Qiu Y., Yang H.-Y., Lu S., Chen W. (2020). A novel hybrid model based on recurrent neural networks for stock market timing. *Soft Computing*.

[42] Singh T., Saxena N., Khurana M., Singh D., Abdalla M., Alshazly H. (2021). Data clustering using moth-flame optimization algorithm. *Sensors*.

[43] Lippi M., Bertini M., Frasconi P. (2013). Short-term traffic flow forecasting: an experimental comparison of time-series analysis and supervised learning. *IEEE Transactions on Intelligent Transportation Systems*.

[44] Long W., Lu Z., Cui L. (2019). Deep learning-based feature engineering for stock price movement prediction. *Knowledge-Based Systems*.

[45] Nahil A., Lyhyaoui A. (2018). Short-term stock price forecasting using kernel principal component analysis and support vector machines: the case of casablanca stock exchange. *Procedia Computer Science*.

[46] Liu W., Pokharel P. P., Principe J. C. (2008). The kernel least-mean-square algorithm. *IEEE Transactions on Signal Processing*.

[47] Liu Z., Loo C. K., Pasupa K. Recurrent kernel online sequential extreme learning machine with kernel adaptive filter for time series prediction.

[48] Hoseinzade E., Haratizadeh S. (2019). CNNpred: CNN-based stock market prediction using a diverse set of variables. *Expert Systems with Applications*.

[49] Yukawa M. (2012). Multikernel adaptive filtering. *IEEE Transactions on Signal Processing*.

[50] Chen B., Zhao S., Zhu P., Príncipe J. C. (2011). Quantized kernel least mean square algorithm. *IEEE Transactions on Neural Networks and Learning Systems*.

[51] Fernandez-Bes J., Elvira V., Van Vaerenbergh S. A probabilistic least-mean-squares filter.

[52] Garcia-Vega S., Zeng X.-J., Keane J. (2018). Stock price prediction using kernel adaptive filtering within a stock market interdependence approach. *SSRN*.

[53] Van Vaerenbergh S., Santamaría I. A comparative study of kernel adaptive filtering algorithms.

[54] Richard C., Bermudez J. C. M., Honeine P. (2008). Online prediction of time series data with kernels. *IEEE Transactions on Signal Processing*.

[55] Zhao S., Chen B., Principe J. C. Kernel adaptive filtering with maximum correntropy criterion.

[56] Liu Z., Loo C. K., Pasupa K., Seera M. (2020). Meta-cognitive recurrent kernel online sequential extreme learning machine with kernel adaptive filter for concept drift handling. *Engineering Applications of Artificial Intelligence*.

[57] Wu X., Chen H., Wang J., Troiano L., Loia V., Fujita H. (2020). Adaptive stock trading strategies with deep reinforcement learning methods. *Information Sciences*.

[58] Liu W., Príncipe J. C. (2008). Kernel affine projection algorithms. *EURASIP Journal on Applied Signal Processing*.

[59] Ahn S. K., Reinsel G. C. (1990). Estimation for partially nonstationary multivariate autoregressive models. *Journal of the American Statistical Association*.

[60] Ouyang T., Huang H., He Y., Tang Z. (2020). Chaotic wind power time series prediction via switching data-driven modes. *Renewable Energy*.

[61] Gao P., Zhang R., Yang X. (2020). The application of stock index price prediction with neural network. *Mathematical and Computational Applications*.

[62] Moghar A., Hamiche M. (2020). Stock market prediction using lstm recurrent neural network. *Procedia Computer Science*.

[63] Nikou M., Mansourfar G., Bagherzadeh J. (2019). Stock price prediction using deep learning algorithm and its comparison with machine learning algorithms. *Intelligent Systems in Accounting, Finance and Management*.

